# Electrophysiological features of repetitive focal Purkinje ventricular arrhythmias originating from the proximal cardiac conduction system

**DOI:** 10.1002/joa3.12787

**Published:** 2022-10-07

**Authors:** Shota Ikeda, Kazuo Sakamoto, Daigo Tokudome, Shunsuke Kawai, Kiyohiro Ogawa, Kazuhiro Nagaoka, Susumu Takase, Shinya Kowase, Yasushi Mukai, Akihiko Nogami, Hiroyuki Tsutsui

**Affiliations:** ^1^ Department of Cardiovascular Medicine, Graduate School of Medical Sciences Kyushu University Fukuoka Japan; ^2^ Department of Cardiology Yokohama Rosai Hospital Yokohama Japan; ^3^ Clinical Engineering Department Yokohama Rosai Hospital Yokohama Japan; ^4^ Department of Cardiology Fukuoka Red Cross Hospital Fukuoka Japan; ^5^ Department of Cardiology Fukuoka City Hospital Fukuoka Japan; ^6^ Department of Cardiology St. Mary's Hospital Fukuoka Japan; ^7^ Department of Cardiology, Faculty of Medicine University of Tsukuba Ibaraki Japan

**Keywords:** cardiac conduction system, catheter ablation, focal Purkinje ventricular tachycardia, non‐reentrant Purkinje ventricular tachycardia, ventricular arrhythmia

## Abstract

**Background:**

Focal Purkinje ventricular arrhythmias (VAs) might originate from the vicinity of the proximal portion of the cardiac conducting system. This study aimed to clarify the features associated with focal Purkinje VAs originating from the proximal conduction system.

**Methods:**

A total of 18 patients with focal Purkinje VAs undergoing radiofrequency catheter ablation (RFCA) were retrospectively examined and divided into the proximal type or the non‐proximal type. The proximal type was defined as having the origin at the proximal half of the interventricular septum, or the proximal half and the septal side of the anterior wall. The 12‐lead electrocardiogram and electrophysiological findings were investigated.

**Results:**

Seven patients met criteria for proximal type of focal Purkinje VA. Out of the 7, 4 patients with proximal VAs had multiple QRS morphologies of VAs clinically, whereas out of 11 patients with non‐proximal VAs, only 1 had multiple morphologies (*p* = .047). VA QRS duration was shorter in the proximal type than in the non‐proximal type (111.2 ± 19.8 ms vs. 135.7 ± 17.7 ms; *p* = .003). The absolute axis difference between sinus rhythm and VA was smaller in the proximal type (80.4 ± 46.1°vs. 138.8 ± 59.6°; *p* = .014). The absolute axis difference ≤134° was useful in distinguishing the two types. Recurrence of VA was recorded in 3 proximal type patients and 3 non‐proximal type patients. No procedure‐related conduction block was observed.

**Conclusion:**

A VA of absolute axis difference ≤134°, and multiple QRS morphologies of clinical VAs indicate a proximal origin. Focal Purkinje VAs from proximal origins can be suppressed by RFCA without severe conduction disturbance.

## Graphical abstract

Focal Purkinje ventricular arrhythmias originate from the vicinity of the proximal portion of the cardiac conducting system. A VA of absolute axis difference ≤134°, and multiple clinical QRS morphologies of clinical ventricular arrhythmias indicate a proximal origin.

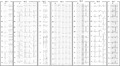

## 1 INTRODUCTION

The Purkinje system is responsible for the mechanism of some ventricular arrhythmias (VAs).^1^ Purkinje‐related monomorphic VAs consist of four subtypes: (a) verapamil‐sensitive left fascicular ventricular tachycardia (VT), (b) Purkinje‐fiber mediated VT post‐infarction, (c) bundle branch reentry and interfascicular reentry VTs, and (d) focal Purkinje VT.^2^ Verapamil‐sensitive left fascicular VT, bundle branch reentry VT, and interfascicular reentry VT have a specific reentrant circuit, and catheter ablation is recommended to suppress them.^3^ Focal Purkinje VTs are uncommon types of VT, and only a few studies have reported the clinical and electrophysiological characteristics of this type of VT.^4^, ^5^ Automaticity or triggered activity of Purkinje fibers are regarded as the cause of focal Purkinje VT. Catheter ablation may be useful to suppress this type of VT, however, it may cause conduction disturbances such as atrioventricular block or left bundle branch block, because of ablation at the proximal site of the conduction system.^2^ Therefore, predicting the origin of VAs before catheter ablation may be important and helpful for successful ablation. The aim of this study is to clarify the features of proximal type of focal Purkinje VAs originating from the vicinity of the proximal cardiac conduction system, and find criteria to distinguish the proximal type from the non‐proximal type.

## 2 METHODS

### 2.1 Study population

A total of 18 patients undergoing radiofrequency catheter ablation (RFCA) were diagnosed with left ventricular focal Purkinje sustained VT, non‐sustained VT (NSVT), and premature ventricular contractions (PVC) from January 2008 to December 2020 in Yokohama Rosai Hospital, Fukuoka Red Cross Hospital, St. Mary's Hospital, Fukuoka City Hospital, and Kyushu University Hospital. All patients had symptomatic VAs. As for PVC, the frequency was more than 10,000 beats per day in all patients except patient 11. PVC in patient 11 is a trigger of sustained ventricular tachycardia. Patient characteristics and electrophysiological features were investigated retrospectively. This study was approved by the local ethics committee at the Kyushu University Hospital (approval No. 21114‐00) and complied with the Declaration of Helsinki. An opt‐out consent was used for this retrospective and noninterventional study.

### 2.2 Diagnosis of focal Purkinje VA

Focal Purkinje VAs were diagnosed based on the following findings. VAs were suppressed with RF application at the site where the earliest preceding Purkinje potentials were recorded during the target VAs. Preceding Purkinje potentials were also recorded during sinus rhythm. The features indicating reentrant ventricular tachycardia were not observed in the cases of sustained VT. In detail, stable sustained tachycardia was induced during catheter ablation session in patients 1, 2, and 14. In patient 2, entrainment pacing was not performed because of completely random RR interval during VT. In other two cases, entrainment pacing was attempted and did not succeed to entrain. In addition, programmed ventricular stimulation never induced sustained ventricular tachycardia. Also, verapamil was not effective in all the cases in which verapamil was administered.^2^

**FIGURE 1:**
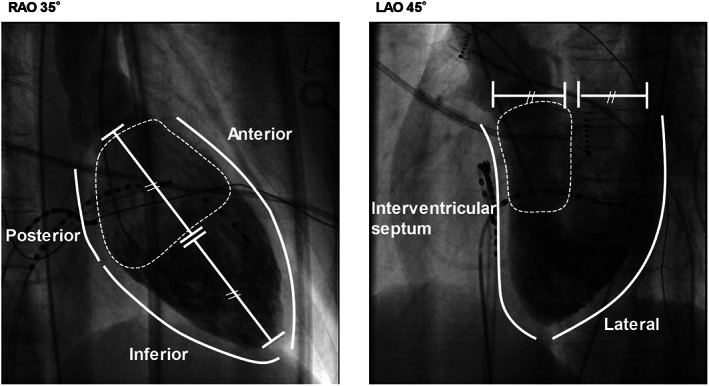
Criteria for proximal and non‐proximal types based on fluoroscopy. The proximal type included those with a successful ablation site at the proximal half portion of the interventricular septum, or the proximal half side and the septal side of the anterior wall based on fluoroscopic imaging; right anterior oblique and left anterior oblique imaging (dotted area).

We divided eligible 18 patients with focal Purkinje VAs into two groups; the proximal type and the non‐proximal type. The proximal type was defined as having a successful ablation site (upon fluoroscopic imaging) located at the proximal half portion of the interventricular septum, or the proximal half side and the septal side of the anterior wall, where the His bundle, left bundle branch, proximal portion of the left anterior fascicle, or the left posterior fascicle were located (Figure 1).

### 2.3 Ablation procedure

In all cases, multipolar electrode catheters were inserted through the femoral vein and placed in the upper right atrium, the His‐bundle region, and the right ventricular apex. Other multipolar electrode catheters were used as needed. A 7‐French non‐irrigated ablation catheter with a 4‐mm tip or 7.5‐French irrigated ablation catheter with a 3.5‐mm tip was inserted via the femoral vein or femoral artery. Radiofrequency (RF) catheter ablation was performed under the guidance of an electroanatomical mapping system. If VAs did not occur spontaneously enough to map, isoproterenol administration, atrial pacing, burst ventricular pacing or ventricular programmed stimulation were performed as appropriate. After induction, the earliest Purkinje potential was mapped. If induction did not produce VAs enough to map, pace mapping was performed. RF energy was applied at the earliest Purkinje potential during VA or the point where the best pace mapping was achieved. The RF power output was titrated to 30 W and a target temperature was 50–60°C without irrigation and 42°C with irrigation for 60–120 s. Junctional contraction and atrioventricular/ventriculo‐atrial conduction were carefully monitored when RF energy was applied near His potential. The origin of the VA was defined as the point where clinical arrhythmias disappeared after RF energy application and there was a lack of VA inducibility after using the same provocation technique as before ablation.

The QRS duration was measured from the earliest deflection from the isoelectric line in any lead to the time of the latest activation in any lead with a paper speed of 100 mm/s. The QRS axes in sinus rhythm and VAs were determined from leads I and aVF. The ECG data were measured with electronic calipers by two investigators (SI and ST) blinded to the site of the origin. The location of the VA's origin was also determined by the same two investigators. Discrepancies were adjudicated by a third investigator (KS).

### 2.4 Statistical analysis

Continuous variables were expressed as mean and standard deviation (SD), and were analyzed using a t‐test. All categorical variables were expressed as raw numbers and compared using the Fisher exact test. To evaluate the axis deviation, the absolute value of difference between the sinus QRS axis and the VA QRS axis (absolute axis difference) was calculated. Logistic regression analyses were employed to elucidate the association between electrocardiogram parameters and proximal VAs. The threshold of absolute axis difference was determined based on the receiver operating characteristic (ROC) curve using the Youden Index. *p* < .05 values were considered statistically significant. All analyses were conducted with JMP 16 (SAS Institute Inc., Cary, NC, USA).

## 3 RESULTS

### 3.1 Clinical characteristics

Eighteen patients were diagnosed with focal Purkinje VA. Tables 1 and 2 shows clinical characteristics. The mean age was 53.7 (range: 18–75), and 11 patients were male. Sustained VTs were clinically observed in seven patients. Five patients had underlying heart diseases (three old myocardial infarctions, one dilated cardiomyopathy, and one hypertrophic cardiomyopathy). In five patients, verapamil was administered but was not effective. Patient 2 had a complete AV block, and a permanent pacemaker was implanted. QRS duration during sinus beats was normal except for patients 1, 10, and 11.

**TABLE 1:** Clinical and electrophysiological characteristics

Clinical and electrophysiological characteristics of patients with proximal type of focal Purkinje ventricular arrhythmias																							
					Sinus beats				Ventricular arrhythmia														
No.	Gener	Age	Clinical VA	Underlying heart disease
QRS duration (ms)	QRS axis (degree)	His‐QRS (ms)	PP–QRS (ms)	QRS duration (ms)	QRS morphology^a^	VA QRS Axis (degree)	Absolute axis difference (degree)	V1 morphology	V6 morphology	His–QRS (ms)	PP–QRS (ms)	Verapamil effect	Effective drug	Induction	Origin	Change of sinus QRS morphology	Conduction Disturbance by RFCA	Outcome
1	M	68	Sustained VT	inf. & ant. MI	155	−30	40	34	146	RBBB + LAD	−56	26	rSR’	Rs	−8	52	No use	Amiodarone	Spontaneous	Basal septum	Yes	No	Recurrence free for 9 months
2	F	48	Sustained VT	‐			‐		74	No BBB and NA	76		rS	Rs	30	68	No effect	‐	Spontaneous	Basal anterior		No	Recurrence free for 77 months
			PVC		RV pacing	‐			101	RBBB + NA	61		rSR’	Rs			Disappeared after elimination of upper VA						
			PVC						85	LBBB + NA	57		QS	R									
3	M	18	Sustained VT	‐	88	95	38	25	123	RBBB + RAD	117	22	rsR’	rS	−22	42	No use	‐	AP	Basal septum	No	No	Recurrence free for 4 months
4	F	34	PVC	‐	98	69	38	24	119	RBBB + LAD	−33	102	rSR’	Rs	0	30	No use	‐	Spontaneous	Basal septum	No	Transient AV prolongation	PVC with another axis was observed 1 day after RFCA
			PVC						111	RBBB + LAD	−48	118	rSR’	Rs			Disappeared after elimination of upper VA						
			PVC						122	RBBB + LAD	−62	131	rSR’	Rs									
			PVC						110	RBBB + NA	15	54	rSR’	Rs									
5	F	69	PVC	‐	79	4	28	17	114	RBBB+LAD	−51	55	R with notch	Rs	Fused	90	No use	‐	Spontaneous	Basal septum	Yes	Transient VA block	PVC with similar morphology was observed 1 day after RFCA
			PVC						116	RBBB+RAD	115	111	R with notch	Rs	0		Disappeared after elimination of upper VA						
			PVC						79	RBBB+RAD	92	88	RS	Rs	−26								
6	F	69	PVC	‐	90	56	37	20	110	RBBB + LAD	−64	120	R with notch	Rs	2	23	No use	‐	Spontaneous	Basal septum	No	Transient LAH	Target PVC was observed at the end of RFCA
			PVC						122	RBBB + LAD	−78	134	R with notch	RS	‐	‐	Disappeared after elimination of upper VA						
7	M	42	PVC	‐	93	88	43	25	136	No BBB and NA	84	4	rS	Rs	−41	24	No use	Bisoprolol, Mexiletine	Spontaneous	Basal anterior	No	Transient RBBB	Recurrence free for 6 months

Abbreviations: Ant., anterior; AP, atrial pacing; BBB, bundle branch block; F, female; Inf., inferior; LAD, left axis deviation; LAH, left anterior hemiblock; LBBB, left bundle branch block; M, male; MI, myocardial infarction; NA, normal axis; PP, Purkinje potential; PVC, premature ventricular contraction; RAD, right axis deviation; RBBB, right bundle branch block; RFCA, radio frequency catheter ablation; RV, right ventricular; VA, ventricular arrhythmia; VT, ventricular tachycardia.

a NA was defined as QRS axis from −30° to +90°. RAD was defined as QRS axis from +90° to ±180°. LAD was defined as QRS axis from −90° to −30°.

**TABLE 2:** Clinical and electrophysiological characteristics of patients with non‐proximal type of focal Purkinje ventricular arrhythmias

No.	Gender	Age	Clinical VA	Underlying heart disease	Sinus beats				Ventricular arrhythmia														
					QRS duration (ms)	QRS axis (degree)	His–QRS (ms)	PP–QRS (ms)	VA QRS duration (ms)	QRS morphology^a^	VA QRS Axis (degree)	Absolute axis difference (degree)	V1 morphology	V6 morphology	His–QRS (ms)	PP–QRS (ms)	Verapamil effect	Effective drug	Induction	Origin	Change of sinus QRS morphology	Conduction disturbance by RFCA	Outcome
8	M	75	Sustained VT	‐	86	68	34	26	106	RBBB+NW	−132	200	rSR’	rS	−24	20	No effect	‐	Burst VP, VPS	Apical septum	No	No	Recurrence free for 108 months
9	M	31	NSVT	‐	97	97	40	17	149	RBBB+NW	−106	203	Notched R	rS	−42	23	No use	‐	Burst VP, VPS	Apical septum	No	No	Recurrence free for 24 months
10	M	61	NSVT	Inf. OMI	102	70	40	29	124	RBBB+LAD	−81	151	R with notch	RS	0	48	No use	Lidocaine	AP, burst VP	Basal inferior	No	No	Recurrence free for 6 months
11	M	57	PVC	DCM	125	61	40	24	158	RBBB+NW	−127	188	R	rS	−100	78	No use	‐	Burst VP, VPS	Apical septum	No	No	PVC with similar morphology was observed 1 day after RFCA
12	M	62	PVC	HCM	88	−24	26	22	134	RBBB+LAD	−66	42	rSR’	rS	−28	24	No use	‐	VPS	Apical septum	Yes	No	PVC with another axis was observed 1 day after RFCA
13	M	69	PVC	‐	94	−5	42	23	136	RBBB+LAD	−82	77	rSR’	RS	−74	52	No use	‐	Burst VP, VPS	Basal inferior	Yes	No	Recurrence free for 17 months
14	F	68	Sustained VT	Inf. OMI	84	46	30	−7	103	RBBB+LAD	−57	103	rSR’	Rs	−12	70	No effect	Lidocaine	Spontaneous	Basal inferior	No	No	Recurrence free for 153 months
15	M	59	PVC	‐	90	23	34	17	145	RBBB+RAD	98	75	R	Rs	−28	20	No use	‐	Spontaneous	Apical septum	Yes	No	Recurrence free for 12 months
16	F	48	PVC	‐	83	42	56	25	130	RBBB+LAD	−66	107	rSR’	RS	Fused	37	No use	‐	Spontaneous	Apical septum	No	No	Recurrence‐free for 72 months after 2nd session for similar PVC
			PVC						147	RBBB+RAD	158	116	rSR’	Rs	‐	‐	Disappeared after elimination of upper VA						
17	M	35	Sustained VT	‐	95	68	69	6	156	RBBB+NW	−150	218	rsR’	rS	−125	37	No effect	‐	Extra pacing from CS ostium	Apical Lateral wall	No	No	Recurrence free for 18 months
18	F	54	PVC	‐	90	97	49	20	140	RBBB+LAD	−89	186	rsR’	rS	−104	37	No effect	Bisoprolol	Spontaneous	Apical septum	No	No	Recurrence free for 36 months

Abbreviations: AP, atrial pacing; CS, coronary sinus; DCM, dilated cardiomyopathy; F, female; HCM, hypertrophic cardiomyopathy; Inf., inferior; LAD, left axis deviation; M, male; NA, normal axis; NSVT, nonsustained ventricular tachycardia; NW, northwest; OMI, old myocardial infarction; PP, Purkinje potential; PVC, premature ventricular contraction; RAD, right axis deviation; RBBB, right bundle branch block; RFCA, radio frequency catheter ablation; RV, right ventricular; VA, ventricular arrhythmia; VP, ventricular pacing; VPS, ventricular programmed stimulation; VT, ventricular tachycardia.

a NA was defined as QRS axis from −30° to +90°. RAD was defined as QRS axis from +90° to ±180°. LAD was defined as QRS axis from −90° to −30°. Northwest axis was defined as QRS axis from −90° to ±180°.

**FIGURE 2:**
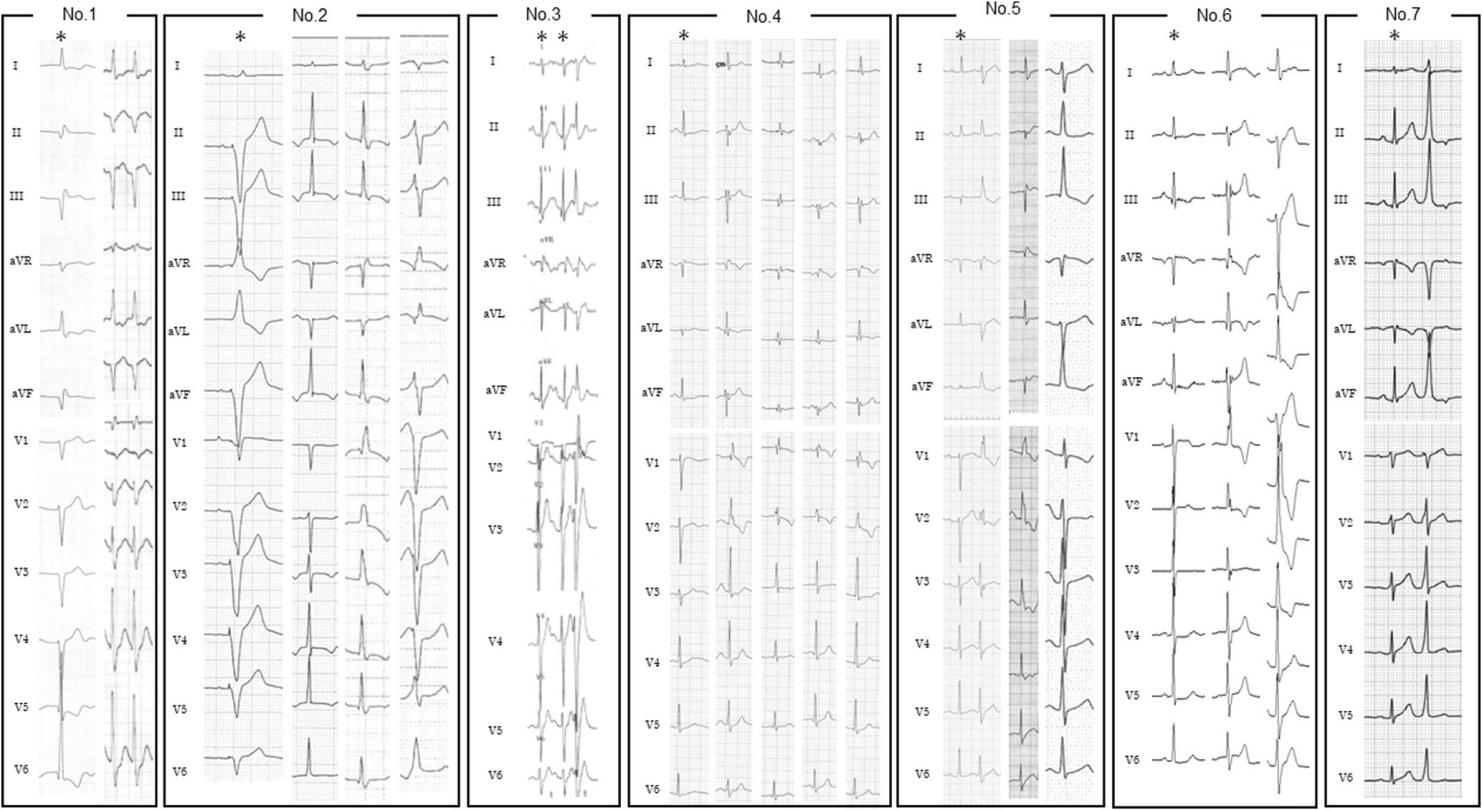
QRS morphologies of proximal focal Purkinje ventricular arrhythmias. Asterisks (*) indicate QRS morphology in sinus rhythm or right ventricular pacing (No. 2).

**FIGURE 3:**
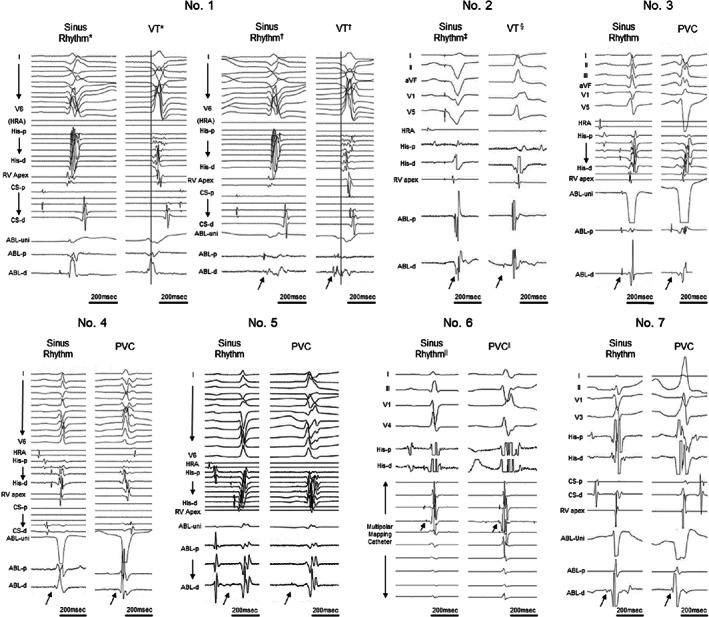
Intracardiac electrocardiogram of proximal type during sinus rhythm and target ventricular arrhythmias. All intracardiac electrocardiograms were recorded at the origins of target ventricular arrhythmias except special notes. Arrows point preceding Purkinje potentials. *Ablation catheter (ABL) was located at the point where His potential was recorded, because His catheter failed to record His potential. Dotted line was set at the QRS onset of V5 for reference. ^†^Ablation catheter (ABL) was located at the origin of the VA. Dotted line was set at the QRS onset of V5 for reference. ^‡^Patient 2 had complete atrioventricular block. Intracardiac electrogram during sinus rhythm was recorded under right ventricular pacing. ^§^The morphology of the target VT was changed by RF application at the non‐origin site near the origin. ^||^The origin of the VA was determined by a multipolar mapping catheter. ABL‐d, distal electrodes of ablation catheter; ABL‐p, proximal electrodes of ablation catheter; ABL‐uni, unipolar electrode of ablation catheter; CS‐d, distal electrodes of coronary sinus catheter; CS‐p, proximal electrodes of coronary sinus catheter; His‐d, distal electrodes of His catheter; His‐p, proximal electrodes of His catheter; HRA, high right atrium; RV, right ventricle.

Of 18 patients with focal Purkinje VAs, 7 patients were classified as those with the proximal type VAs, and 11 patients were classified as those with the non‐proximal type VAs. All QRS morphologies of proximal focal Purkinje VAs are presented in Figure 2, and intracardiac electrocardiograms of proximal type VAs during sinus rhythm and target VAs are shown in Figure 3. A summary of the electrocardiogram findings is presented in Tables 1, 2 and 3. QRS duration of the sinus beat was comparable between the proximal and the non‐proximal type of focal Purkinje VAs. Four patients with the proximal type presented with multiple VA QRS morphologies clinically from a single origin, but only 1 out of 11 patients with the non‐proximal type presented with multiple VA QRS morphologies (*p* = .047). The VA QRS duration was 111.2 ± 19.8 ms (range: 74–146 ms) in the proximal type and 135.7 ± 17.7 ms (range: 103–158 ms) in the non‐proximal type (*p* = .003). One QRS morphology presented left bundle branch block (LBBB) pattern, and two QRS morphologies presented neither right bundle branch block (RBBB) pattern nor left bundle branch block pattern in the proximal type. In the non‐proximal type, all QRS morphologies presented RBBB pattern. No QRS morphology in the proximal type presented northwest axis (QRS axis from −90° to ±180°) deviation whereas 4 QRS morphologies in the non‐proximal type presented northwest axis deviation. The absolute axis difference was 80.4 ± 46.1° in the proximal type and 138.8 ± 59.6° in the non‐proximal type (*p* = .014). His–QRS onset interval during VA was significantly smaller in the proximal type than the non‐proximal type (−8.3 ± 20.1 ms vs −53.7 ± 43.6 ms, *p* = .011). Preceding Purkinje potential–QRS onset during VA was not significantly different between 2 VA types. The QRS morphology of sinus beats could be changed upon RF application for both proximal and non‐proximal types. Clinically significant complications such as permanent atrioventricular block or LBBB did not occur whereas transient conduction disturbances were observed during RFCA in four cases of proximal VAs. We obtained acute procedural success in all cases except patient 6. During follow‐up, 4 out of 7 patients of proximal type and 8 out of 11 patients of non‐proximal type did not have VA recurrence, including VAs seemingly related to clinical VA though presenting other morphologies (*p* = .63).

**TABLE 3:** Summary of electrocardiographic parameters

(A) Analysis by origin			
	Proximal type (*n* = 7 patients)	Non‐proximal type (*n* = 11 patients)	*p* value
Sinus QRS duration, ms	93.3 ± 21.5	93.1 ± 11.7	.98
His potential–QRS onset interval during sinus rhythm, ms	37.3 ± 5.0	41.8 ± 12.3	.41
PP–QRS onset interval during sinus rhythm, ms	24.2 ± 5.8	18.4 ± 10.4	.23
Multiple VA QRS morphology, *n*	4/7	1/11	.047

(B) Analysis by morphology			
	Proximal type (*n* = 15 VA QRS morphologies)	Non‐proximal type (*n* = 12 VA QRS morphologies)	*p* value
VA QRS duration, ms	111.2 ± 19.8	135.7 ± 17.7	.003
Northwest axis in VA QRS, *n*	0/15	4/12	.028
VA axis, degree	15.0 ± 73.3	−58.3 ± 92.5	.030
Absolute value of axis difference, degree	80.4 ± 46.1	138.8 ± 59.6	.014
His potential–QRS onset during VA, ms	−8.3 ± 20.1	−53.7 ± 43.6	.011
PP–QRS onset during VA, ms	47.0 ± 24.9	40.5 ± 19.8	.55

Abbreviations: PP, Purkinje potential; VA, ventricular arrhythmia.

### 3.2 Predictive factors for proximal type

Logistic regression analyses were performed to identify predictive factors for proximal VAs (Table 4). In univariate analyses, absolute axis difference (odds ratio [OR]: 0.98, 95% CI: 0.96–1.00, per 1° increase, *p* = .031) and VA QRS duration (OR: 0.93, 95% CI: 0.87–0.98, per 1 ms increase, *p* = .014) were significantly associated with the proximal type. In multivariate analyses, absolute axis difference was significantly associated with the proximal type (OR: 0.97, 95% CI: 0.94–1.00, per 1° increase, *p* = .043). VA QRS duration tended to be associated with the proximal type (OR: 0.90, 95% CI: 0.81–1.00, per 1 ms increase, *p* = .053).

**TABLE 4:** Predictive factors for proximal ventricular arrhythmias

	Univariate analysis		Multivariate analysis	
	OR (95% CI)	*p* value	OR (95% CI)	*p* value
Absolute axis difference (per 1° increase)	0.98 (0.96–1.00)	.031	0.97 (0.94–1.00)	.043
VA QRS duration (per 1 ms increase)	0.93 (0.87–0.98)	.014	0.90 (0.81–1.00)	.053

*Note*: Univariate and multivariate logistic regression analysis for the proximal type of VA was performed.

Abbreviations: CI, confidence interval; OR, odds ratio; VA ventricular arrhythmia.

**FIGURE 4:**
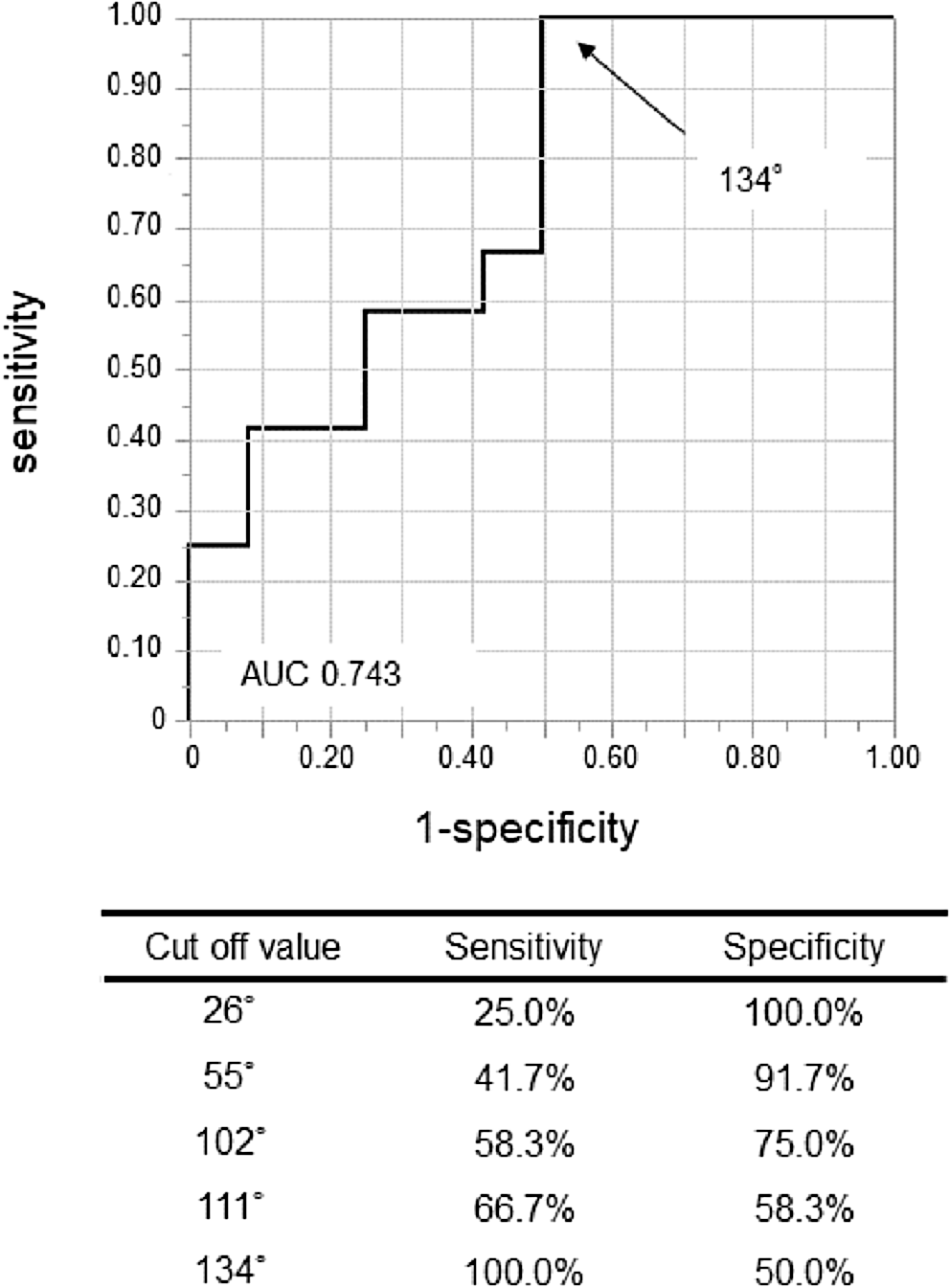
Receiver operating characteristic (ROC) curve analyses for the absolute axis difference predicting the proximal type of focal Purkinje VA. The ROC curve analysis of the absolute axis difference revealed a cutoff value of 134° for the proximal type, with sensitivity at 100.0% and specificity at 50.0%. The area under the curve was 0.743.

ROC curve analysis was performed to evaluate the predictive value of the absolute axis difference, wherein the best thresholds were 134° (sensitivity: 100.0%, specificity: 50.0%) with AUC = 0.743 (Figure 4).

## 4 DISCUSSION

This study could demonstrate electrophysiological characteristics of focal Purkinje VAs originating from the vicinity of the proximal portion of the cardiac conduction system. The proximal type of focal Purkinje VAs demonstrated a shorter QRS duration and multiple QRS morphologies in more cases, and a smaller deviation in QRS axis from that of the sinus rhythm.

### 4.1 Multiple VA QRS morphologies in proximal type of focal Purkinje VAs

In the proximal type of focal Purkinje VA, multiple VA QRS morphologies were clinically recorded, seemingly originating from a single region. On the other hand, the non‐proximal type only presented multiple VA QRS morphologies only in 1 case in the studied patients. A possible reason for multiple VA QRS morphologies in the proximal type could be multiple origins of excitation. However, VAs with multiple morphologies simultaneously disappeared after RF application to the site where major VA QRS morphology was eliminated in our several cases. Therefore, it is conceivable that multiple QRS morphologies could be exerted by a single origin in the proximal type of focal Purkinje VA. This may be caused by conductance changes in the proximal portion of His‐Purkinje system and surrounding myocardial tissue during VA. A similar phenomenon was reported in other variations of VA originating from the papillary muscles or from the outflow tract.^6^, ^7^, ^8^ Although the possibility of accidental elimination of other origins which caused VAs with other QRS morphology could not be completely denied, we have already reported that RF application near the origin changed the QRS morphology and axis drastically.^9^ Therefore, RF application could change VA QRS morphology with completely different axis. Recently, case series analyzing VAs involving the left septal fascicule reported that multiform and narrow QRS complex VA could indicate an involvement of the left septal fascicule.^10^ This might be another possible mechanism.

Only one case in proximal type of VAs presented with LBBB morphology (patient 2). She did not have a history of myocardial infarction or other underlying heart diseases other than complete atrioventricular block. The VA presented narrow QRS duration of 105 ms and was eliminated by the RF energy application to the area of proximal anterior fascicle. In addition, no radiofrequency energy was applied to other sites including right ventricular wall. Considering these facts, we assumed that VAs including VA with LBBB pattern originated from single origin rather than multiple origins. The reason for LBBB might be spontaneously delayed conduction of left bundle branch.

### 4.2 Duration and axis deviation of QRS in the proximal type of focal Purkinje VA

Focal Purkinje VAs analyzed in this study showed relatively shorter QRS duration. In particular, the proximal type presented with significantly shorter QRS duration compared to the non‐proximal type. Although VA QRS duration in patient 1 was 146 ms, he had a previous myocardial infarction and the QRS duration in sinus rhythm was 155 ms. The shorter QRS duration in the proximal type may be because of an efficient propagation using the proximal portion of the cardiac conduction system. A shorter interval between the VA QRS onset and the His potential seemed to support this. Although it did not reach statistical significance, shorter QRS duration tended to predict proximal type of VA.

Proximal focal Purkinje VAs never presented with a northwest QRS axis (−90°to ±180°), which is one of the characteristics of ventricular tachycardia,^11^, ^12^, ^13^ whereas four cases of the non‐proximal type did. We hypothesized that the QRS axis of proximal focal Purkinje VAs should be less deviated compared to that of the non‐proximal type. The axis of QRS could be deviated leftward or rightward, thus comparison of mean value of axis of QRS seemed to be less meaningful. Instead, the absolute axis difference, i.e., the difference between the sinus and VA axes, was analyzed in this study, and it was significantly smaller in the proximal type compared to the non‐proximal type. A proximal VA conducts from base to apex and from septal side to lateral side (similar to sinus beats) whereas the non‐proximal type may originate from the apical or lateral portions. The ROC curve analysis revealed that the absolute axis difference with a cutoff of 134° may be useful to distinguish the proximal from the non‐proximal type.

### 4.3 Procedural success rate and complications

In our case series, 3 out of 7 proximal cases and 3 out of 11 non‐proximal cases had recurrence, and the result was similar to the report by Ahmed et al. that 4 out of 15 cases had recurrence.^5^ Severe conduction disturbance, which could occur because of RF catheter ablation especially at the vicinity of the proximal conduction system, is the serious complication to avoid.^2^, ^14^ Permanent atrioventricular block or LBBB were not observed in our studied patients. However, transient AV interval prolongation, transient ventriculo‐atrial block, transient left anterior hemiblock, and transient RBBB were observed, and sufficient RF energy could not be applied in such cases. This limited energy application might be related relatively high recurrence rate in the proximal cases.

Recently, PVC originating from the proximal left anterior fascicle (LAF) is reported to be successfully eliminated from the right coronary cusp (RCC), however, left anterior hemiblock was observed in one out of eight cases with approach from the RCC.^15^ Although we actually recorded the local potentials from coronary cusps in some cases, RF energy was not applied at coronary cusps because the local potentials were not the earliest. Thus, while approach from the RCC might be useful and important option to eliminate VAs from the LAF, a special caution to avoid conduction disturbance are required.

### 4.4 Limitations

We acknowledge the limitations of this study. First, this study is a small retrospective case series and has heterogeneity of the population. Evidently, some VAs might originate from Purkinje fibers and the others from left anterior or posterior fascicle in various underlying heart disease. Second, proximal and non‐proximal types are defined based on fluoroscopic images. This definition might be useful for planning therapeutic strategy, however, that did not consider electrophysiological characteristics such as local or fascicular potentials. Especially, from the electrophysiological perspectives, the distances from the proximal portion of the left bundle branch to the origin of VA might be more suitable. In this study, these electrophysiological analyses could not be performed because of some missing data. However, the interval between VA QRS onset and retrograde His potential was significantly shorter in the proximal group. This supports that the origin of VA in the proximal group was closer to the His bundle than the VA in the non‐proximal group. Although this study revealed features of focal Purkinje VAs from the vicinity of the proximal portion of the cardiac conduction system, larger studies are needed to investigate this uncommon type of VA.

## 5 CONCLUSION

A smaller axis deviation and multiple VA QRS morphologies are the features of the proximal type of focal Purkinje VA originating from the vicinity of the proximal cardiac conduction system. An absolute axis difference of ≤134° are indicators of proximal origin. Catheter ablation was similarly effective for both proximal and non‐proximal types. These features would be helpful in the diagnosis and treatment of focal Purkinje VAs.

## FUNDING INFORMATION

This research did not receive any specific grant from funding agencies in the public, commercial, or not‐for‐profit sectors.

## CONFLICT OF INTEREST

Shinya Kowase has received honoraria from Medtronic, Abbott, Japan Lifeline, Boston Scientific, and BIOTRONIK. Akihiko Nogami has received lecture honoraria from Abbott and an endowment from Medtronic. All other authors have no conflicts of interest to declare.

## ETHICS APPROVAL STATEMENT

This study was approved by the local ethics committee at the Kyushu University Hospital (approval No. 21114‐00) and complied with the Declaration of Helsinki. An opt‐out consent was used for this retrospective and noninterventional study.

## Data Availability

All data generated or analyzed during this study are included in this published article.
